# Tissue clearing and quantitative confocal microscopy reveal significant impairment of cerebrovascular structure and function following moderate prenatal alcohol exposure

**DOI:** 10.21203/rs.3.rs-11011631/v1

**Published:** 2026-10-19

**Authors:** Gabriela Perales, Marissa R. Westenskow, Robert J. Oliver, Amy S. Gardiner

## Abstract

Fetal alcohol spectrum disorders (FASD) occur after prenatal alcohol exposure (PAE) and include a wide range of neurocognitive deficits potentially resulting from abnormal cerebrovascular development and the expression of factors regulating vessel formation and function. We previously determined that upregulation of miR-150-5p within brain microvascular endothelial cells (BMVECs) during PAE affects pathways required for angiogenesis, vascular development, and blood-brain barrier (BBB) integrity. Using a moderate PAE mouse model, we examined the structure and integrity of the cortical microvasculature at embryonic day 18 (E18). We used CLARITY, a tissue clearing and imaging technique allowing for high-quality quantitative analysis of the microvasculature. We also used fluorescent tracers to examine BBB integrity *in vivo* . In addition, we assessed microvascular structure and gene expression in embryonic cortices following miR-150-5p inhibition. We found that E18 PAE cortices displayed significant differences in microvascular structure compared to control cortices. We also found that E18 PAE cortices were significantly more permeable to dextran tracers compared to controls. miR-150-5p inhibition rescued specific PAE-induced microvascular defects. MiR-150-5p inhibition also resulted in elevated expression of genes involved in angiogenesis, BBB maintenance, and vascular development. Overall, we show that moderate PAE adversely affects cerebrovascular structure and BBB function in the developing brain.

